# Publication quality 2D graphs with less manual effort due to explicit use of dual coordinate systems

**DOI:** 10.1186/1751-0473-9-22

**Published:** 2014-10-21

**Authors:** Daniel A Wagenaar

**Affiliations:** 1 Department of Biological Sciences, University of Cincinnati, PO Box 210006, Cincinnati, OH 45221, USA

**Keywords:** Data plotting, Typography, Matlab

## Abstract

Creating visually pleasing graphs in data visualization programs such as Matlab is surprisingly challenging. One common problem is that the positions and sizes of non-data elements such as textual annotations must typically be specified in either data coordinates or in absolute paper coordinates, whereas it would be more natural to specify them using a combination of these coordinate systems. I propose a framework in which it is easy to express, e.g., “this label should appear 2 mm to the right of the data point at (3, 2)” or “this arrow should point to the datum at (2, 1) and be 5 mm long.” I describe an algorithm for the correct layout of graphs of arbitrary complexity with automatic axis scaling within this framework. An implementation is provided in the form of a complete 2D plotting package that can be used to produce publication-quality graphs from within Matlab or Octave.

## Background

Computer programs for the two-dimensional graphical display of numerical data abound (e.g., Gnuplot [1], Igor Pro [2], Matlab [3], Octave [4]). Such programs commonly allow specification of data in arbitrary coordinates, and will automatically make sensible choices for axis ranges and many other aspects of the visualization. Rarely however, are the results immediately usable for professional publication. Less than elegant automatic positioning of text labels is especially common. While manual fine tuning is possible, this quickly becomes laborious even for relatively simple graphs, and in any case requires great attention to visual detail from users who would probably rather concentrate on their science. Why is correct positioning of text labels in graphs so challenging? Consider a typical task: placement of a textual annotation by a data point in an x-y graph. The text should appear centered a little below the data point. How much is a little? Probably a millimeter or two, but you cannot tell that to Matlab (or Gnuplot, etc.). Instead, you have to specify the label location in data coordinates. By trial and error, the user can experiment and find that in one particular graph the annotation should be placed at (1, 0.95) and in another at (1, 0.6) to appear at the desired distance below the data point at (1, 1). This will depend on the range of the graph’s axes, and the scale at which the graph is rendered. As an alternative, the graph could be exported and postprocessed in a graphics program such as Inkscape [5] or Illustrator [6], but producing consistent results this way is difficult, and it becomes laborious when many similar graphs are to be made. Wouldn’t it be convenient to simply be able to specify: “the top of this label should go 1.5 mm below the data point (1, 1)” from within the program that generates the graph in the first place?

A related, but not identical, problem is the determination of appropriate axis ranges when text labels may extend beyond the data range. If the final size of the graph is prespecified, then it is usually necessary to manually shrink the output from Matlab posthoc to make such labels fit. Naturally, that affects font sizes, which then need to be corrected, which typically makes it necessary to move the labels a little bit, and so on.

Here, I describe a program for generating two-dimensional graphs that explicitly acknowledges the relevance of two complementary coordinate systems: the logical coordinates of the data, and the physical coordinates of the output medium. As a result, text annotations can be positioned in the most natural way, and scaling the data axes to make the graph and its annotations fit in a prespecified area can be automated. The program can be used stand-alone, or—more conveniently—from within Matlab or Octave.

## Implementation

### Notation and statement of the problem

Data coordinates will be denoted in lower case as (*x,y*). Paper coordinates will be denoted in upper case as (*X,Y*). Although arbitrary affine transformations between data coordinates and paper coordinates can be supported with little additional complexity, in this paper I will assume that data are placed on the paper with only scaling and translation, so that we can write

$$\begin{array}{lcrr} X & = & \text{ax}+b & \\ Y & = & \text{cy}+\mathrm{d.} & \end{array}$$

The location of graphical elements may be written as a 4-tuple (*x*,*y*,*δX*,*δY*) specifying a point displaced by (*δX*,*δY*) paper units from the data point (*x*,*y*). This simple formalism is more flexible than it might seem at first glance: It can, for instance, readily handle rotated text, which would have (*δX*,*δY*) be a function of the rotation angle, the point size, the font, and the text itself, but—importantly—not of the transformation parameters (*a, b, c, d*). However, this formalism cannot handle situations where the displacement *is* a function of the transformation parameters, as would be the case, e.g., when rotating text to parallel a curve specified in data coordinates. More on this later.

The problem to be solved is to find a set of transformation parameters (*a, b, c, d*) that makes all graphical elements fit within a predetermined area of size (*W, H*) on the paper and that causes all of the available space to be filled.

### Linear programming?

In mathematical terms, this problem may be restated as maximizing *a* and *b* under the constraints that for the bounds of each graphical element:

$$\begin{array}{lcrr} 0 & \leq & \text{ax}+b+\delta X\leq W & \\ 0 & \leq & \text{cy}+d+\delta Y\leq H. & \end{array}$$

In principle, this problem could be solved directly by linear programming [7]. However, for a practical implementation in software, an alternative approach was found to be preferable, not just because it was easier to program, but also because it naturally circumvented the limitation noted above.

### Shrink-to-fit algorithm

The algorithm arrives at the optimal solution in a number of iterations. In the first iteration, the bounding box (*x*_0_,*y*_0_) – (*x*_1_,*y*_1_) of the data is determined, and a transformation matrix is derived to fit this bounding box onto the destination area on the paper. The transformation parameters (*a*,*b*,*c*,*d*) follow trivially from demanding that (*x*_0_,*y*_0_) gets mapped to the origin on the paper while (*x*_1_,*y*_1_) gets mapped to (*W, H*):

$$\begin{array}{lcrr} a & = & \frac{W}{x_{1}-x_{0}} & \\ b & = & -{\text{ax}}_{0}, & \end{array}$$

with analogous expressions for the vertical axis. (For brevity, only the expressions for the horizontal axis placement will be given in the following. The expressions for the vertical axis are always directly analogous).

Next, all graph elements that are specified by a location displaced from given data points are considered. Take, for instance, a text label that is (*w,h*) points large as rendered and that is to be placed a distance (*δX*,*δY*) away from data point (*x*,*y*). This label would have a bounding box (*X*_0_,*Y*_0_) – (*X*_1_,*Y*_1_), where *X*_0 _= *ax *+ *b *+ *δX* and *X*_1 _= *ax *+ *b *+ *δX *+ *w*.

If the bounding boxes for all graph elements fall within the destination area, the algorithm is done. Otherwise, the transformation matrix needs to be modified to shrink the graph. Define *ΔL*, *ΔR*, *ΔT*, and *ΔB* to be the amounts by which the union of all bounding boxes protrudes outside the destination area to the left, right, top, and bottom respectively, and let *ΔL* (*ΔR*, etc.) be zero if the corresponding edge does not protrude.

A naive way to correct for the protusions is to replace:

(1) $$a\leftarrow \frac{W-\Delta R-\Delta L}{x_{1}-x_{0}}$$

and then, using the new value for *a*:

(2) $$b\leftarrow -{\text{ax}}_{0}+\Delta L.$$

**Figure 1:**
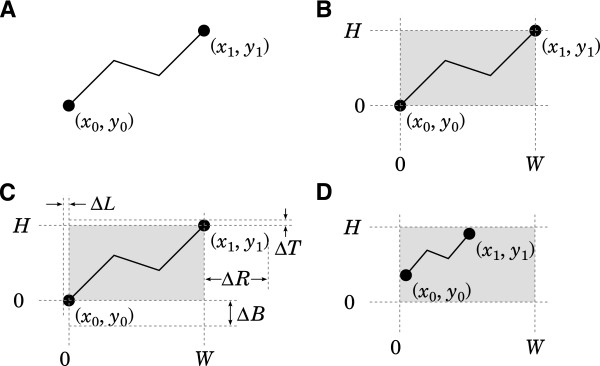
**Laying out a simple graph. A**. The graph to be laid out. The text labels “ (*x*_0_,*y*_0_)” and “ (*x*_1_,*y*_1_)” are to be understood as part of the layout. **B**. If the graph is to fit within the gray rectangle, it is not sufficient to map *x*_0_ to *X*_0_ and *x*_1_ to *X*_1_. **C**. That is because such a mapping would make several graphical elements protrude past the desired bounding box. **D**. After shrinking per (1) and (2), the graph does fit as designed. Note that font sizes and line widths are not affected by the shrinking operation.

**Figure 2:**
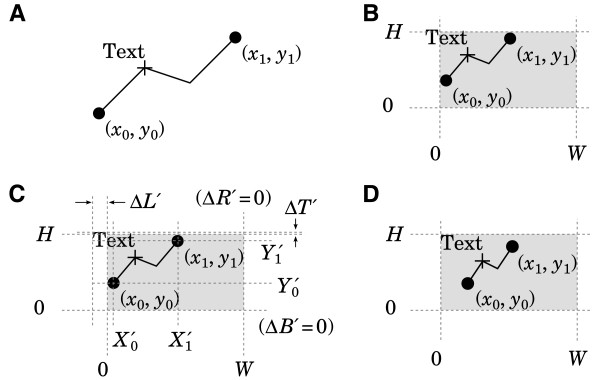
**Laying out a more complex graph. A**. The label “Text” is attached to the data point marked with a “+” sign. **B**. Applying a shrink step makes the label protrude. **C**. A second shrink step is needed to shift the ends of the data range farther inward. **D**. After several steps, the desired result is obtained.

In the simplest case (Figure 1), this actually ensures that previously protruding labels now fall within the originally desired rectangle. However, labels that are attached to data points in the middle of the graph may be *caused* to protrude by this step (Figure 2). The solution is to consider the points $\left(X_{0}^{\prime },Y_{0}^{\prime }\right)$ and $\left(X_{1}^{\prime },Y_{1}^{\prime }\right)$ that the original data range gets mapped to by shrinking according to the naive procedure above (Figure 2C) and then to re-apply the equivalent of (1) and (2) to shift these points by *ΔL*^′^ and *ΔR*^′^ respectively: 

$$a\leftarrow \frac{(X_{1}^{\prime }-X_{0}^{\prime })-\Delta R^{\prime }-\Delta L^{\prime }-2\varepsilon }{x_{1}-x_{0}}$$

followed by

$$b\leftarrow -{\text{ax}}_{0}+X_{0}^{\prime }+\Delta L^{\prime }+\varepsilon .$$

**Figure 3:**
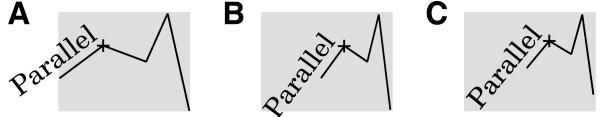
**Correct layout of a graph with displacements that depend on the transformation parameters. A**. The label “Parallel” is anchored to the data point marked with a “+” sign and rotated to be parallel with the preceding segment of the curve. After initial layout, the label protrudes to the left. **B**. It is not enough to shrink only in the horizontal dimension, as the steeper orientation of the label makes it protrude below the destination area. **C**. Fortunately, the iterative algorithm automatically detects this and shrinks the graph also in the vertical dimension.

Here *ε* is a small user-defined constant, e.g., *ε *= 0.25 pt (0.1 mm), to compensate for the fact that text anchored to a point *x *> *x*_0_ would otherwise be moved somewhat less than *ΔL*^′^ by the above substitution. After several iterations, this leads to the correct result (Figure 2D). As an interesting aside, this iterative algorithm even works when the displacement of a graphical element is itself a function of the transformation parameters, as is the case with text placed parallel to a data curve (Figure 3).

It should be noted that not all layout challenges *have* solutions. For instance, if the label in Figure 2 had been “Impossible” rather than “Text,” no amount of shrinking would have allowed it to fit together with the label “ (*x*_1_,*y*_1_)” attached to the rightmost data point. In such instances, *a* becomes negative after a number of iterations, at which point the algorithm simply has to give up.

### Plotting package

A layout algorithm by itself is not practically usable, so I implemented the above algorithm in conjunction with a 2D graph plotting package, named QPlot. The core of the program was written in C++ [8] using the Qt library [9] to achieve operating system independence. This program and its user manual are available at http://www.danielwagenaar.net/qplot. The program may be used stand-alone which is expecially useful for automated graph generation. In addition, a library of Matlab/Octave functions may be used to conveniently graph data produced within these popular scientific computation environments with QPlot. (QPlot could not have been implemented directly in Matlab or Octave, because these languages do not permit graphical elements to be accurately measured).

## Results and discussion

**Figure 4:**
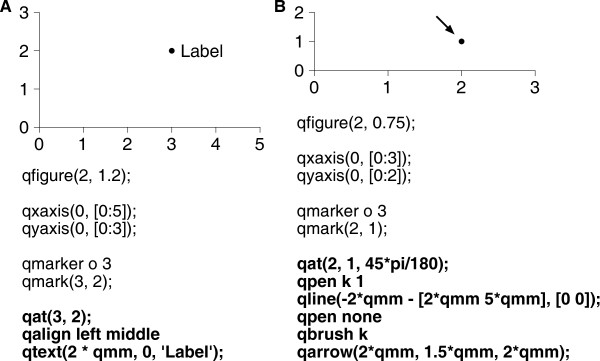
**Examples from the abstract. A**. The “text label” example. **B**. The “arrow” example. Key passages of the code are set in bold face. Note how the angle of the arrow is 45° even though the x- and y-axes have a different scale.

QPlot was designed to produce elegant figures with relative ease. For instance, the examples presented in the Abstract are shown in Figure 4. The “text label” example (Figure 4A) mostly speaks for itself, except for the phrase “2 * qmm”, which means “2 mm”. (Internally, QPlot uses points (1/72 inch) for its paper units). The “arrow” example (Figure 4B) is slightly more involved, because the original formulation of the problem left several key parameters unspecified: the angle at which the arrow points (45°), the distance between the tip of the arrow and the data point (2 mm), the width of the line (1 point), and the size of the arrowhead (2 × 1.5 mm).

**Figure 5:**
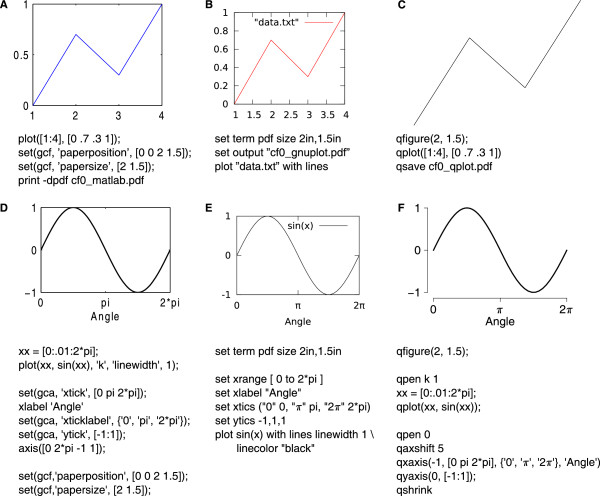
**Comparison with Matlab and Gnuplot. A–C**: Minimal working examples in Matlab **(A)**, Gnuplot **(B)**, and QPlot **(C)** to produce a pdf file of a simple graph. **D–F**: Demonstration of basic axis labeling in Matlab **(D)**, Gnuplot **(E)**, and QPlot **(F)**. The code to produce each example is reproduced below each graph.

**Figure 6:**
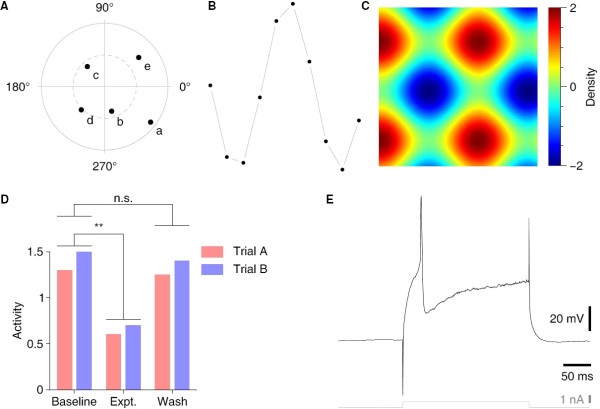
**Several more examples. A**. A circular plot with annotated points. **B**. An example of connecting lines that do not touch the data points. **C**. An example of an image map. **D**. A bar graphs with indicators of statistical significance. (Fictional data.) **E**. A trace from an electrophysiological recording from a neuron. The code for these examples is available at http://www.danielwagenaar.net/qplot.

A comparison with two other popular plotting programs, Matlab and Gnuplot, is given in Figure 5. Note that QPlot does not venture to automatically guess axis limits or tick intervals, since in most real-world situations these would have to be fine-tuned anyway. However, pressing “c” in QPlot’s main window displays the graph coordinates of the mouse pointer and pressing “r” displays a set of basic rulers. These can be used as aids in determining appropriate values. (As an aside, note how much cleaner the graph in Figure 5F looks thanks to the slightly displaced axes [10]).Several further real-world examples are presented in Figure 6. A tutorial with further examples and a reference guide with examples using each of the available commands are available at the project’s homepage.

## Conclusion

A new representation for laying out visualizations of scientific data has been presented that explicitly acknowledges the existence of two complementary sets of coordinates: paper coordinates (measured in millimeters or inches) and data coordinates (measured in arbitrary units). Using these dual coordinate systems, describing the placement of non-data elements [11] in appropriate locations relative to the data becomes much more straightforward. As a result, the placement of text labels, axes, and other elements can be guaranteed (within certain limits) to remain correct irrespective of the scaling of the data axes. This dual representation also enabled the formulation of a layout algorithm that automatically scales a graph to fit the available space while respecting constraints on the placement of text and other elements (Figures 1, 2 and 3).

To ensure that the results of this study are practically usable, a 2D graph plotting package was written that implements the dual representation and the automatic scaling algorithm. This software, QPlot, can be used from within the popular Matlab/Octave environments and is freely available online.

While the representation introduced in this article was described in terms of two-dimensional graphs and the current version of QPlot likewise only produces two-dimensional graphs, extension to three-dimensional data coordinates is in principle straightforward and may be implemented in a future version of the software.

## Availability and requirements

**Project name:** QPlot**Project home page:**http://www.danielwagenaar.net/qplot**Project archive:**http://www.launchpad.net/qqplot**Operating systems:** QPlot has been tested on Linux. A binary version is available for Ubuntu 14.04. QPlot should compile from available sources on MacOS and Windows and wherever Qt and Octave are available.**Programming languages:** C++, Matlab**Other requirements:** QPlot needs the Qt libraries, version 4.8 or later. QPlot needs either Matlab, version 7 or later, or Octave, version 3.6 or later.**License:** GNU General Public License ver. 3+**Any restrictions to use by-non-academics:** None

## Competing interests

The author declares that he has no competing interests.

## Authors’ contributions

DAW conceived of the algorithms, implemented the software, and wrote the paper.

## Authors’ information

DAW is an Assistant Professor at the Department of Biological Sciences at the University of Cincinnati. Previously, he was a Senior Research Fellow at the California Institute of Technology.
